# Isolation and Characterization of a Glycosyl Hydrolase Family 16 β-Agarase from a Mangrove Soil Metagenomic Library

**DOI:** 10.3390/ijms17081360

**Published:** 2016-08-19

**Authors:** Zhimao Mai, Hongfei Su, Si Zhang

**Affiliations:** Key Laboratory of Tropical Marine Bio-Resources and Ecology, Guangdong Key Laboratory of Marine Materia Medica, South China Sea Institute of Oceanology, Chinese Academy of Sciences, Guangzhou 510301, China; maizhimao@scsio.ac.cn (Z.M.); suhongfei17@126.com (H.S.)

**Keywords:** agarase, metagenomic library, agar, neoagaro-oligosaccharides

## Abstract

A mangrove soil metagenomic library was constructed and a β-agarase gene designated as *AgaML* was isolated by functional screening. The gene encoded for a 659-amino-acids polypeptide with an estimated molecular mass of 71.6 kDa. The deduced polypeptide sequences of *AgaML* showed the highest identity of 73% with the glycoside hydrolase family 16 β-agarase from *Microbulbifer agarilyticus* in the GenBank database. *AgaML* was cloned and highly expressed in *Escherichia coli* BL21(DE3). The purified recombinant protein, AgaML, showed optimal activity at 50 °C and pH 7.0. The kinetic parameters of *K*_m_ and *V*_max_ values toward agarose were 4.6 mg·mL^−1^ and 967.5 μM·min^−1^·mg^−1^, respectively. AgaML hydrolyzed the β-1,4-glycosidic linkages of agar to generate neoagarotetraose (NA4) and neoagarohexaose (NA6) as the main products. These characteristics suggest that AgaML has potential application in cosmetic, pharmaceuticals and food industries.

## 1. Introduction

Agar is an important polysaccharide that consists of agarose and agaropectin. Agarose has a linear chain structure of alternating residues of 3-*O*-linked β-d-galactopyranose and 4-*O*-linked 3,6-anhydro-α-l-galactopyranose [1]. For the stabilizing properties and gelling ability of agarose, it is widely applied in cosmetics, pharmaceuticals and food industries [2]. However, due to the high viscosity, low water solubility and undigested characteristic, its application value has not been fully developed. Instead, agaro-oligosaccharides exhibit not only excellent dissolubility and easily absorbed, but also physiological activity, such as anticancer, anti-oxidation, anti-inflammation, antivirus and immune enhancement [3,4,5,6]. Recently, there is increasingly interest in conversion of agar into agaro-oligosaccharides. Compared with traditional technologies of acid hydrolysis, enzymatic conversion of agar into agaro-oligosaccharides has the advantages of mild reaction conditions, high efficiency and specificity, simple processing and being environmentally friendly. Thus, it is considered to be a feasible approach with broad prospects for the development of novel drugs and functional foods.

Agarases that catalyze the hydrolysis of agarose into oligosaccharides have been divided into two classes based on their hydrolysis sites. The α-agarases (E.C. 3.2.1.158) hydrolyze α-1,3-linkages in agarose to generate agaro-oligosaccharides, while the β-agarases (E.C. 3.2.1.81) hydrolyze β-1,4 linkage in agarose to form neoagaro-oligosaccharides [7,8]. Most agarases currently studied and applied are β-agarases. On the basis of amino acid sequences homology in the carbohydrate-active enzymes (CAZY) database, β-agarases are classified into four families: glycoside hydrolases family 16 (GH16), GH50, GH 86 and GH118 [9]. To date, many β-agarases have been isolated from various bacteria, such as from the genera *Pseudomonas*, *Agarivorans*, *Vibrio*, *Alteromonas* and *Bacillus* [10,11,12,13,14]. However, these reported agarases were still not satisfactory due to their low catalytic activity, thermal stability and productivity. Thus, screening and isolation of novel agarases from microorganisms is urgently in demand.

Current estimates indicate that less than 1% of microorganisms are readily culturable with known cultivation techniques [15]. The traditional method for microbial enzyme mining is based on the culturable microorganisms, which may result in the loss of major portions of the microbial communities. Metagenomics circumvent the traditional cultivation, and directly isolate and clone the target gene from the environmental microorganism DNA. It provides an effective approach for mining novel biocatalysts from uncultured microorganism. Many novel genes encoding different enzymes and secondary metabolites have been isolated from microbial communities without cultivation, such as cellulase [16], lipase [17], protease [18], amylase [19], etc. However, to our knowledge, no agarase has been isolated and characterized from metagenomic library, except for several putative agarase genes identified from a soil metagenome [20].

In this study, we exploited the metagenome for isolation of genes encoding β-agarases. The detailed DNA sequence of the selected β-agarase was analyzed. The recombinant β-agarase was purified and characterized for the further study.

## 2. Results and Discussion

### 2.1. Construction of Metagenomic Library and Isolation of Agarase Gene

The size and quantity of DNA extracted from the mangrove soil was met the requirement of fosmid library construction. The crude extracted metagenomics DNA was then purified using pulsed-field gel electrophoresis (PFGE). Finally, a fosmid library with 100,000 clones was constructed. Restriction analysis of randomly selected clones showed that the DNA fragments insert sizes ranged from 20 to 55 kb and the average size was about 30 kb. The total insertion DNA of the fosmid library was estimated as more than 3 Gb [21]. The positive fosmid clones were identified when they generated pits after incubation. Three independent clones with agarase activity from the fosmid library were obtained. After enzymatic activity analysis, one clone with highest agarase activity was selected and the recombinant plasmid in this clone was designated as Fos84. Restriction analysis of Fos84 showed that the inserted DNA fragment was approximately 31 kb (date not shown). One agarase-producing clone was identified by screening the subclone library. The plasmid from positive clones was sequenced. A single open-reading frame encoded agarase gene, designated *AgaML*, was obtained.

The currently reported agarases are mostly isolated from marine bacteria. Mangrove is a unique ecosystem that possesses both terrestrial ecosystem and marine ecosystem characteristics. However, there has not yet been agarase isolated from the mangrove environment. The metagenomic approach has been widely used for exploiting novel enzymes [22]. But for agarase, only one study reported that four clones encoding 12 putative agarase genes were identified by activity-based screening from a soil metagenome [20]. In this study, three independent clones with agarase activity were isolated, which exhibited lower hit rate of positive clone.

### 2.2. Sequence Analysis

**Figure 1:**
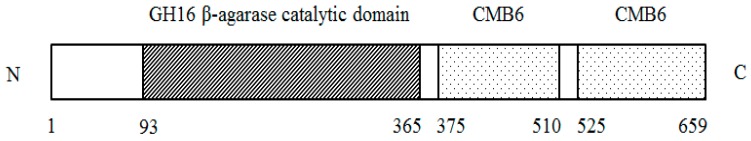
Schematic overview of the domain structure in AgaML. The amino acid numbers that refer to each module are indicated.

Sequence analysis revealed that the gene *AgaML* consists of 1980 bp with the overall G + C content of 55%. It encoded a protein, designated AgaML, with 659 amino acids. The estimated molecular mass of AgaML was 71.6 kDa and the isoelectric point (pI) was 5.05. Analysis of SignalP 4.1 server (http://www.cbs.dtu.dk/services/SignalP/) revealed that there was no signal peptide in AgaML. According to the NCBI search program of conserved-domain database, a β-agarase catalytic domain of the glycoside hydrolases family 16 (GH16) and two carbohydrate-binding modules (CBM6) were found (Figure 1). The CBM6 modules were found in many glycoside hydrolases, including xylanases, endoglucanase and mannanases, which were described as binding to xylan, cellulose, mixed β-(1,3)(1,4)-glucan and β-1,3-glucan [23,24,25,26]. However, the deletion of one or two CBM6 modules in AgaML had no effect on the catalytic activity and stability (data not shown). It was reported that CBM6 modules in GH16 β-agarases functioned as special targets to the non-reducing end of agarose chains [27,28].

**Figure 2:**
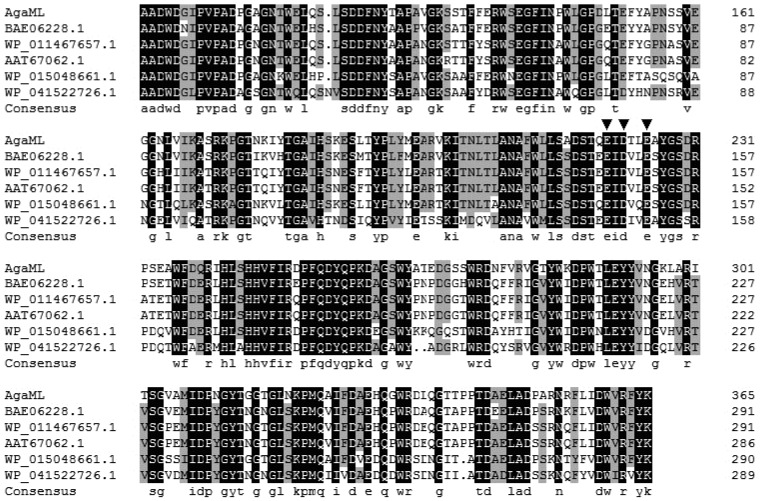
Multiple sequence alignments of the catalytic domain in AgaML with other known β-agarases belonging to glycoside hydrolases family 16 (GH16). **BAE06228.1:** β-agarase from *Microbulbifer agarilyticus* JAMB A3; **WP_011467657.1:** β-agarase from *Saccharophagus degradans*; **AAT67062.1:** β-agarase from *Saccharophagus degradans* 2-40; **WP_015048661.1:** β-agarase from *Simiduia agarivorans*; **WP_041522726.1:** β-agarase from agarase *Gilvimarinus agarilyticus*. The predicted active site residues of AgaML (Glu-220, Glu-225 and Asp-222) are represented as solid inverted triangle symbol.

The deduced protein of AgaML showed high identity with β-agarases in the NCBI database: 73% to the β-agarase from *Microbulbifer agarilyticus* (GenBank accession no. BAE06228.1), 71% to the β-agarase from *Saccharophagus degradans* 2-40 (GenBank accession no. AAT67062.1), 66% to the β-agarase from *Saccharophagus* sp. AG21 (GenBank accession no. AFR90184.1) and 66% to the β-agarase from *Simiduia* sp. TM-2 (GenBank accession no. BAQ95400.1). Multiple sequence alignments of the GH16 β-agarase catalytic domain in AgaML with these β-agarases were observed and the catalytic residues were also predicted (Figure 2). Based on the sequence similarities analysis of AgaML with the known GH16 family β-agarases, we assumed that the active site of AgaML were Glu-220 (as the nucleophile) and Glu-225 (as the acid/basic) [29]. Another conserved acidic amino acid residue Asp-222 might be important for maintaining the charge in the environment of the catalytic amino acids.

**Figure 3:**
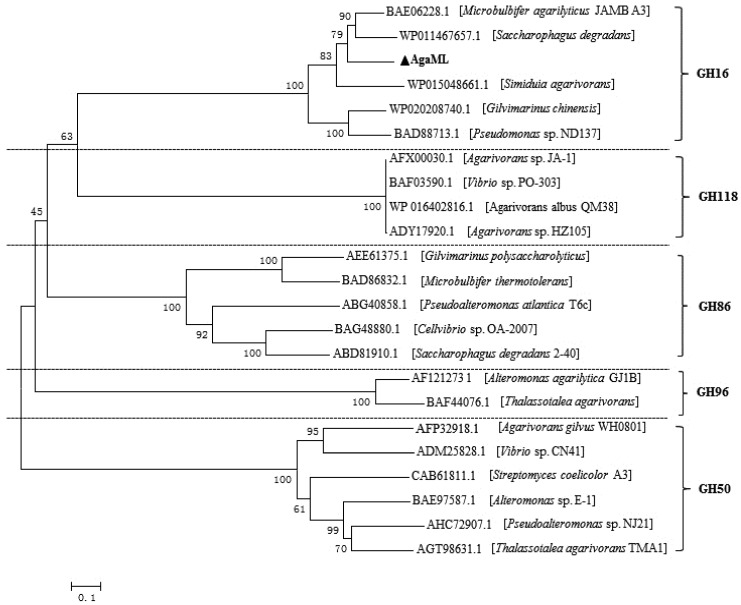
Phylogenetic tree analysis of AgaML with other known agarases based on the amino acid sequences. The AgaML is shown with bold solid triangle. All of the agarase sequences are grouped into five families by the carbohydrate-active enzymes (CAZY) database. Their accession numbers and original genus are indicated on the tree. The phylogenetic tree was constructed using MEGA (Version 5.0) with the neighbor-joining method and 1000 bootstrap replications are indicated at branching points.

To analyze the relationship of AgaML with the known β-agarase members from various species, a phylogenetic tree was constructed (Figure 3). The selected agarases were comprised into five clades, represented by GH16, GH50, GH86, GH96 and GH118, respectively. AgaML was grouped into GH16.

### 2.3. Expression and Purification of Recombination Agarase

**Figure 4:**
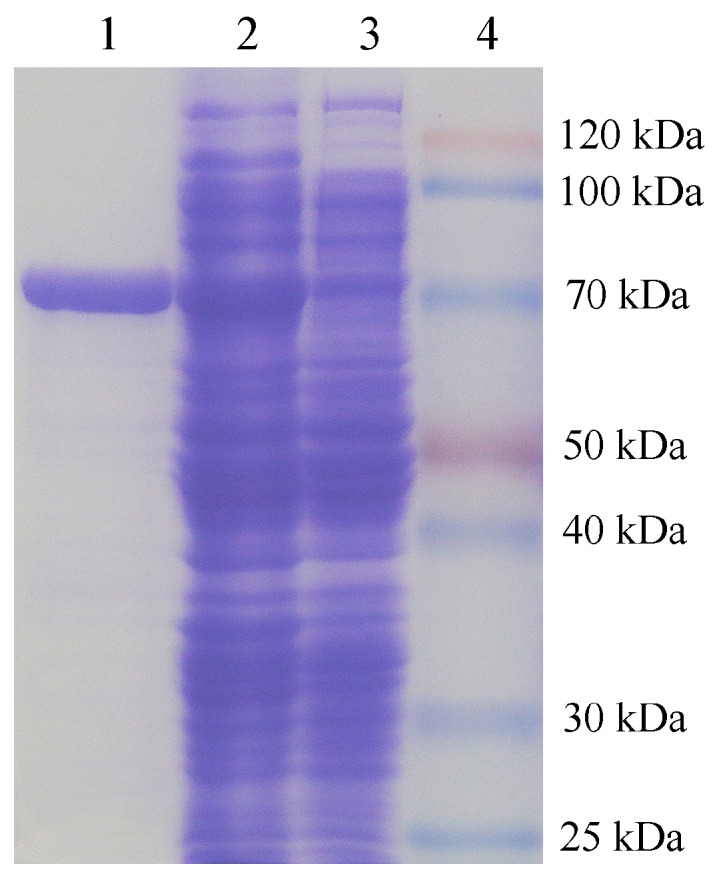
10% sodium dodecyl sulfate polyacrylamide gel electropheresis (SDS-PAGE) analysis of AgaML. (**1**) Purified AgaML; (**2**) Recombinant *Escherichia coli* BL21(DE3) cells harboring pET-*AgaML* after induced; (**3**) Uninduced recombinant *Escherichia coli* BL21(DE3) cells harboring pET-*AgaML*; and (**4**) Protein marker.

The β-agarase gene *AgaML* was cloned into pET22b(+) vector, and then transformed into *Escherichia coli* BL21(DE3) cells. The agarase activity was detected in the recombinant *Escherichia coli* BL21(DE3) cells after isopropyl-β-d-galactopyranoside (IPTG) induction. The crude β-agarase AgaML was purified by Ni^2+^-NTA affinity chromatography. The purified AgaML was analyzed using sodium dodecyl sulfate polyacrylamide gel electropheresis (SDS-PAGE). A single band with an apparent molecular mass of 71.6 kDa corresponding to the calculated size of AgaML was observed (Figure 4). The AgaML exhibited maximum catalytic activity of 967.5 μM·min^−1^·mg^−1^ and a *K*_m_ of 4.6 mg·mL^−1^ for agarose on the optimal condition. The kinetic parameters of *K*_m_ and *V*_max_ for different agarases are various. Most reported agarases exhibited a *K*_m_ between 1 and 50 mg·mL^−1^, and a *V*_max_ between 10 and 1000 μM·min^−1^·mg^−1^ [27,30,31,32,33,34,35]. AgaML displayed a relatively higher specific activity than most of the reported agarases.

### 2.4. Effects of pH and Temperature on the Activity of Recombination Agarase

**Figure 5:**
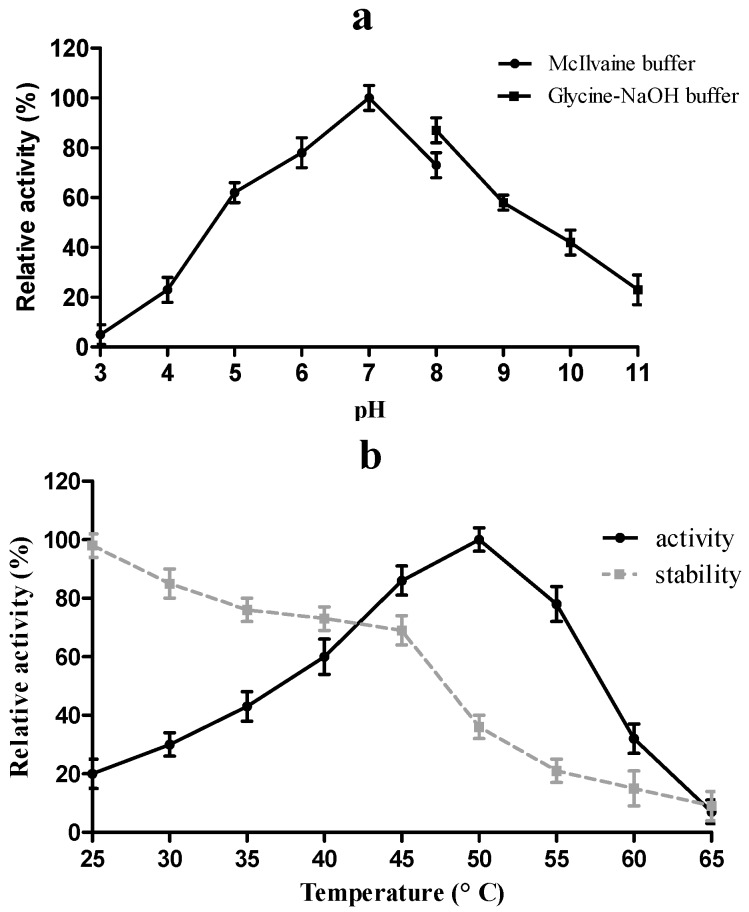
Effects of pH and temperature on the activity of AgaML. (**a**) Effect of pH on enzyme activity was measured in 0.05 M McIlvaine buffer with pH range from 3.0 to 11.0; and (**b**) Effect of temperature on enzyme activity were measured at temperatures ranging from 25 to 65 °C. Thermo stability analysis was observed after preincubation at temperatures ranging from 25 to 65 °C for 1 h. The error bars represent the means ± standard deviation (SD) (*n* = 3).

The effects of pH and temperature on the activity of AgaML were measured (Figure 5). The maximum activity of AgaML was observed at pH 7.0 and 50 °C. It displayed high thermostability at the temperature below 45 °C, which retained more than 60% activity after incubation for 1 h (Figure 5b). Similar to most of the reported β-agarases, AgaML exhibited optimal temperature at >40 °C, which was higher than the gelling temperature of agar (~38 °C) [36]. The high catalytic activity and thermostability at temperatures above the gelling temperature could offer advantage for enzymatic conversion of agar or marine algae into oligosaccharide.

### 2.5. Effects of Various Metal Ions and Reagents on the Activity of Recombination Agarase

The effects of different metal ions and reagents on AgaML activity were measured at pH 7.0 and 50 °C in the presence of the tested metal ions and reagents. As shown in Table 1, no significant effects on the activity of AgaML was observed in Na^+^, K^+^, Fe^3+^ and Co^2+^; however, 1 mM metal ions (Mn^2+^, Ca^2+^ and Ba^2+^) had a slight positive effect on the AgaML activity. However, the AgaML activity was inhibited by Mg^2+^, Zn^2+^, Cu^2+^ and Cd^2+^, and its activity was also inhibited by 10 mM chelator (EDTA) and detergent (SDS). As reported, metal ions commonly existing in seawater, including Na^+^, K^+^, Fe^3+^, Ca^2+^, Mn^2+^ and Ba^2+^, had no significant inhibition in many agarase while Cu^2+^ was a potential inhibitor to most agarase [27,30,37]. The AgaML activity was inhibited significantly by EDTA suggested that it might be a metal ion-dependent enzyme.

### 2.6. Analysis of the Hydrolysis Pattern and Products of Recombination Agarase

**Figure 6:**
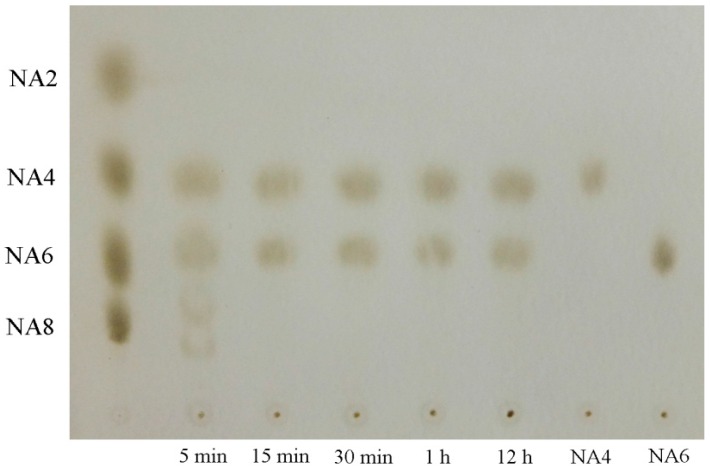
Thin layer chromatography (TLC) analysis of agar degradation by AgaML at different time points. Hydrolysis reactions were measured at pH 7.0 and 50 °C. Hydrolysates were taken at different incubation times and analyzed by TLC. Neoagarobiose (NA2), neoagarotetraose (NA4), neoagarohexaose (NA6), and neoagarooctaose (NA8) were used as standards.

To determine the hydrolysis type and products of AgaML on agar, the hydrolysates at different reaction times were identified by thin layer chromatography (TLC). The result (Figure 6) showed that the AgaML hydrolyzed agar to generate neoagaro-oligosaccharides with various degrees of polymerization (DPs) during the initial stage of the reaction. This hydrolysis pattern suggested that AgaML was an endo-type β-agarase. The final products of the enzyme reaction after prolonged incubation were neoagarotetraose (NA4) and neoagarohexaose (NA6). When neoagarotetraose and neoagarohexaose were used as substrates hydrolyzed by AgaML, no hydrolysis products were observed.

The hydrolysis products of different agarases toward agar are varied, but the same GH family agarases exhibit similar digestion pattern. The hydrolysis products of reported agarases toward agar are listed in Table 2 (these are not comprehensive but represent a selection of previous studies). Generally, the main products toward agar are NA4 and NA6 by agarases of the GH16 family, NA2 or NA4 by those of GH50 family, and NA6, neoagarooctaose (NA8) or higher degrees of polymerization of neoagaro-oligosaccharides by agarases of GH86 family. The main products were NA4 and NA6 by AgaML (Figure 6), indicating it belonged to GH16 family.

## 3. Materials and Methods

### 3.1. Strains, Plasmids, and Culture Conditions

The *Escherichia coli* EPI300-T1^R^ strain served as host and Copycontrol pCC2FOS (Epicentre, Madison, WI, USA) served as vector for metagenomic library construction. The *Escherichia coli* DH5α and *Escherichia coli* BL21(DE3) (Novagen, Madison, WI, USA) strains served as the hosts for gene cloning and expression, respectively. The *Escherichia coli* strains were cultured at 37 °C in Luria-Bertani (LB) broth. The plasmids pMD18-T (Takara, Kyoto, Japan) and pET22b(+) (Novagen) were used for cloning and expression vectors, respectively. All restriction enzymes, ligases, *Ex* Taq^TM^ DNA polymerase and related reagents were purchased from Takara. The standards neoagarobiose (NA2), neoagarotetraose (NA4), neoagarohexaose (NA6), and neoagarooctaose (NA8) were from Qingdao BZ Oligo Biotech Co., Ltd., Qingdao, China. All other chemicals used were of analytical grade.

### 3.2. Construction of Metagenomic Library

The topsoil (0–10 cm) was sampled from Mangrove Reserve of Sanya City (18°15′16.32″ N, 109°30′28.10″ E), Hainan Province, China. The DNA extraction from soil samples was conducted based on direct lysis methods with minor modifications [48]. The soil DNA was purified by pulsed-field gel electrophoresis (PFGE) and sheared to approximately 40 kb fragments. The blunt-ended DNA was ligated to the cloning-ready Copycontrol pCC2FOS vector, and then packaged and plated on EPI300-T1^R^ cells. The constructed libraries were collected in 96-well plates and stored at −80 °C.

### 3.3. Library Screening and AgaML Gene Isolation

The metagenomic library clones in 96-well plates were cultured onto Luria-Bertani (LB) agar supplemented with 25 μg/mL of chloramphenicol at 37 °C for 16 h. The positive clones showing pits were selected and then stained by Lugol’s iodine solution (containing 5% I_2_ and 10% KI). The clones with agarolytic activity were visualized as clear zones on a brown background. The plasmids of positive clones from fosmid library were extracted d and digested with restriction enzymes *Sau*3AI. The DNA fragments with the size ranging from 3 to 9 kb were recovered, purified and ligated to *Bam*H I digested pUC19 vector. The ligated mixture was transformed into *Escherichia coli* DH5α for subcloned library construction. Clones with agarolytic activity were selected and the plasmids from positive clones were sequenced.

### 3.4. Sequence Analysis and Classification of AgaML

The sequence similarities and the conserved domain search were performed by BLAST program (http://www.ncbi.nlm.nih.gov/BLAST). The signal peptide sequence prediction was performed using SignalP 4.1 Server (http://www.cbs.dtu.dk/services/SignalP/). Multiple sequence alignment was conducted using ClustalW (http://www.ch.embnet.org/software/ClustalW.html) and DNAMAN (Version 6.0, Lynnon, San Ramon, CA, USA). A phylogenetic tree was created using MEGA 5.0 software (http://www.megasoftware.net/) with the neighbor-joining (NJ) method.

### 3.5. Cloning and Expression of AgaML

The *AgaML* gene was amplified from the plasmid of positive clone by using the primer pair of aga-F: 5′-GCATGCCATGGATGTGTATCCACCTTCAATCCCCTACCG-3′ and aga-F: 5′-GCACGCGGATCCGGTTGGCTGTGAGGACTAATCTGTCCAG-3′. The nucleotides underlined in the primers aga-F and aga-F indicated *Nco* I and *Bam*H I digestion site, respectively. The PCR product was purified and digested with *Nco* I and *Bam*H I, and then ligated into *Nco* I-*Bam*H I site of expression vector pET22b(+).The recombinant plasmid, designated as pET-*AgaML*, was transformed into *Escherichia coli* BL21(DE3). The transformed *Escherichia coli* BL21(DE3) cells were cultured in LB medium supplemented with ampicillin (100 μg/mL) at 37 °C. When the optical density value at 600 nm reached 0.6, the cells were induced with 1 μM (final concentration) isopropyl-β-d-galactopyranoside (IPTG) and further cultured at 22 °C for 16 h.

### 3.6. Purification of Recombinant Agarase

The induced *Escherichia coli* BL21(DE3) cells were harvested by centrifugation (6000 rpm, 15 min, 4 °C), washed twice with Tris-HCl buffer (pH 7.5), and disrupted on ice by sonication. The AgaML was purified with Ni^2+^-NTA chromatography. The purified recombinant protein was analyzed by SDS-PAGE. The concentration of purified protein was measured by the Bradford method [49].

### 3.7. Agarase Activity Assay

The agarase activity was determined using 3,5-dinitrosalicylic acid (DNS) method [50]. The reaction mixure contained 1 μL diluted enzyme solution (0.8 μg of purified agarase), 199 μL of McIlvaine buffer (0.05 M, pH 7.0) and 1% (*w*/*v*) agarose. After incubation at 50 °C for 15 min, the reaction was terminated with 200 μL DNS and then boiled for 10 min. The heat-inactivated recombinant β-agarase served as a negative control. The absorbance was measured at 540 nm and values for reducing sugar were expressed as d-galactose equivalents. Agarase activity (U) was defined as the amount of enzyme that produced 1 μM of reducing sugar per min under the assay conditions.

### 3.8. Effects of pH and Temperature on Recombinant Agarase Activity

The effect of pH on AgaML activity was assayed in 0.05 M McIlvaine buffer (pH 3.0–8.0) and 0.05 M glycine-NaOH buffer (pH 8.0–11.0) at 50 °C. The effect of temperature on AgaML activity was detected at different temperatures (25 °C–65 °C). The thermostability of AgaML was evaluated by determining the residual activity of AgaML after preincubation at different temperatures ranging from 25 to 65 °C for 1 h.

### 3.9. Effects of Various Metal Ions and Reagents on Recombinant Agarase Activity

The sensitivity of AgaML to various metal ions, denaturants and chelators were analyzed by measuring the enzyme activity supplemented with different concentrations of Na^+^, NH_4_^+^, K^+^, Mg^2+^, Zn^2+^, Ca^2+^, Ba^2+^, Cu^2+^, Co^2+^, Cd^2+^, Fe^3+^, EDTA and SDS. All enzyme activities were determined in three independent experiments. The relative activity was expressed as the percentage of activity respect to that determined under the standard condition without metal ions, denaturants and chelators.

### 3.10. Hydrolysis Products Analysis of Recombinant Agarase

The hydrolysis products of AgaML towards to agar were determined by thin-layer chromatography (TLC) [43]. The hydrolysis reaction containing purified AgaML and 0.5% agar in McIlvaine buffer (0.05 M, pH 7.0). After incubating for different times at 50 °C, the reaction was stop by incubation in a boiling water bath for 10 min and the inactivation enzyme was removed by centrifuging at 4 °C for 20 min. The reaction mixture was spotted on silica gel 60 TLC plates (Merck, San Diego, CA, USA). The plates were developed with *n*-butanol-acetic acid–water (1:2:1, *v*/*v*/*v*) solution and then immersed rapidly in 10% H_2_SO_4_ (*v*/*v*). The oligosaccharides were visualized by heating the plates at 90 °C. Neoagarobiose (NA2), neoagarotetraose (NA4), neoagarohexaose (NA6), and neoagarooctaose (NA8) were used as standards.

### 3.11. Nucleotide Sequence Accession Number

The *AgaML* gene nucleotide sequence reported was deposited in the GenBank database under accession numbers KX388156.

## 4. Conclusions

A β-agarase gene *AgaML* was isolated from a mangrove soil metagenomic library for the first time. The recombination β-agarase AgaML exhibited high catalytic activity toward agarose. It hydrolyzed agar to generate neoagarotetraose and neoagarohexaose as the main products. Moreover, AgaML displayed optimal temperature higher than the gelling temperature of agar, and it was stable at temperatures below 45 °C. Most common metal ions also had no significant inhibition on AgaML activity. These characteristics indicate that AgaML is a good candidate for industrial applications. This study also highlights the utility of metagenomic approach in discovering novel β-agarase for conversion agar into neoagaro-oligosaccharides.

## Figures and Tables

**Table 1:** Effects of various metal ions and chemical reagents on the activity of AgaML.

Reagents	Concentration (mM)	Relative Activity (%) *^a^*
None	-	100.0 ± 4.2 *^b^*
Na^+^	100	103 ± 3.4
K^+^	100	104 ± 3.5
NH_4_^+^	100	69 ± 5.6
Mn^2+^	1	115 ± 4.3
Mg^2+^	1	87 ± 4.6
Fe^3+^	1	98 ± 3.2
Zn^2+^	1	74 ± 6.2
Ca^2+^	1	108 ± 6.4
Cu^2+^	1	75 ± 5.1
Ba^2+^	1	113 ± 4.2
Co^2+^	1	103 ± 3.6
Cd^2+^	1	85 ± 4.3
EDTA	10	17 ± 5.7
SDS	10	73 ± 5.3

***^a^*** Assay was measured at the optimum conditions; *^b^* Values represent the means ± standard deviation (SD) (*n* = 3); - Standard condition without metal ions, chelators or denaturants.

**Table 2:** Hydrolysis products of characterized agarases.

Family	Protein	Strain	Products	References
GH16	AgaML	Metagenomic library	NA4, NA6	This study
	AgaA	*Pseudoalteromonas* sp. CY24	NA2, NA4, NA6	[38]
	AgaG1	*Alteromonas* sp. GNUM1	NA2, NA4	[39]
	AgaA	*Agarivorans* sp. LQ48	NA4, NA6	[27]
	AgaYT	*Flammeovirga yaeyamensis*	NA2, NA4	[40]
GH50	RagaA11	*Agarivorans* sp. JAMB-A11	NA2	[41]
	Unnamed	*Agarivorans* sp. JA-1	NA2, NA4	[11]
	AgWH50A	*Agarivorans gilus* WH0801	NA4	[42]
GH86	AgaP4383	*Flammeovirga pacifica* WPAGA1	NA4, NA6	[37]
	AgaO	*Microbulbifer* sp. JAMB-A94	NA6	[43]
	AgaA	*Cellvibrio* sp. OA-2007	NA2, NA4	[44]
GH118	AgaXa	*Catenovulum* sp. X3	NA6, NA8, NA10, NA12	[45]
	Agarase-a	*Agarivorans albus* OAY02	NA2, NA4, NA6	[46]
	AgaB	*Pseudoalteromonas* sp. CY24	NA8, NA10	[47]
